# Thermophotovoltaic efficiency of 40%

**DOI:** 10.1038/s41586-022-04473-y

**Published:** 2022-04-13

**Authors:** Alina LaPotin, Kevin L. Schulte, Myles A. Steiner, Kyle Buznitsky, Colin C. Kelsall, Daniel J. Friedman, Eric J. Tervo, Ryan M. France, Michelle R. Young, Andrew Rohskopf, Shomik Verma, Evelyn N. Wang, Asegun Henry

**Affiliations:** 1 Department of Mechanical Engineering, Massachusetts Institute of Technology, Cambridge, MA USA; 2 National Renewable Energy Laboratory, Golden, CO USA

**Keywords:** Devices for energy harvesting, Solar energy and photovoltaic technology, Energy storage

## Abstract

Thermophotovoltaics (TPVs) convert predominantly infrared wavelength light to electricity via the photovoltaic effect, and can enable approaches to energy storage^1,2^ and conversion^3–9^ that use higher temperature heat sources than the turbines that are ubiquitous in electricity production today. Since the first demonstration of 29% efficient TPVs (Fig. 1a) using an integrated back surface reflector and a tungsten emitter at 2,000 °C (ref. ^10^), TPV fabrication and performance have improved^11,12^. However, despite predictions that TPV efficiencies can exceed 50% (refs. ^11,13,14^), the demonstrated efficiencies are still only as high as 32%, albeit at much lower temperatures below 1,300 °C (refs. ^13–15^). Here we report the fabrication and measurement of TPV cells with efficiencies of more than 40% and experimentally demonstrate the efficiency of high-bandgap tandem TPV cells. The TPV cells are two-junction devices comprising III–V materials with bandgaps between 1.0 and 1.4 eV that are optimized for emitter temperatures of 1,900–2,400 °C. The cells exploit the concept of band-edge spectral filtering to obtain high efficiency, using highly reflective back surface reflectors to reject unusable sub-bandgap radiation back to the emitter. A 1.4/1.2 eV device reached a maximum efficiency of (41.1 ± 1)% operating at a power density of 2.39 W cm^–2^ and an emitter temperature of 2,400 °C. A 1.2/1.0 eV device reached a maximum efficiency of (39.3 ± 1)% operating at a power density of 1.8 W cm^–2^ and an emitter temperature of 2,127 °C. These cells can be integrated into a TPV system for thermal energy grid storage to enable dispatchable renewable energy. This creates a pathway for thermal energy grid storage to reach sufficiently high efficiency and sufficiently low cost to enable decarbonization of the electricity grid.

## Abstract (web summary)

Two-junction TPV cells with efficiencies of more than 40% are reported, using an emitter with a temperature between 1,900 and 2,400 °C, for integration into a TPV system for thermal energy grid storage.

## Main

Here we report TPV efficiency measurements of more than 40%, determined by simultaneous measurement of electric power output and heat dissipation from the device by calorimetry. This record  experimental demonstration of TPV efficiency was enabled by (1) the usage of higher bandgap materials in combination with emitter temperatures between 1,900 and 2,400 °C, (2) high-performance multi-junction architectures with bandgap tunability enabled by high-quality metamorphic epitaxy^16^ and (3) the integration of a highly reflective back surface reflector (BSR) for band-edge filtering^11,13^.

**Fig. 1:**
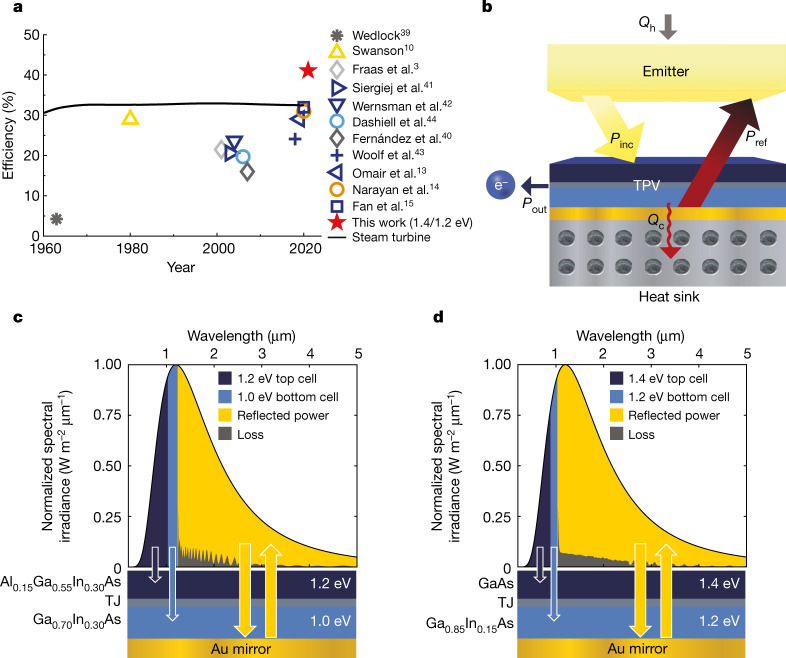
Tandem TPVs. **a**, History of some TPV efficiencies^12^ with different cell materials: Ge^39,40^ (dark grey), Si^10^ (yellow), GaSb^3^ (light grey), InGaAs^13,15,41–43^ (dark blue), InGaAsSb^44^ (light blue) and GaAs^14^ (orange). The black line shows the average thermal efficiency of power generation in the United States using a steam turbine (coal and nuclear)^36,37^. Before the year 2000, turbine efficiencies shown also include natural gas. **b**, Energy that is incident on the TPVs (${P}_{{\rm{inc}}}$) can be converted to electricity (${P}_{{\rm{out}}}$), reflected back to the emitter (${P}_{{\rm{ref}}}$) or thermalized because of inefficiencies in the cell and back reflector (${Q}_{{\rm{c}}}$). **c**, **d**, The 1.2/1.0 eV (**c**) and 1.4/1.2 eV (**d**) tandems that were fabricated and characterized in this work, and a representative spectrum shape at the average emitter temperature (2,150 °C blackbody) indicating the spectral bands that can be converted to electricity by the top and bottom junction of a TPV cell. A gold mirror on the back of the cell reflects approximately 93% of the below bandgap photons, allowing this energy to be recycled. TJ represents the tunnel junction.

**Extended Data Fig. 1:**
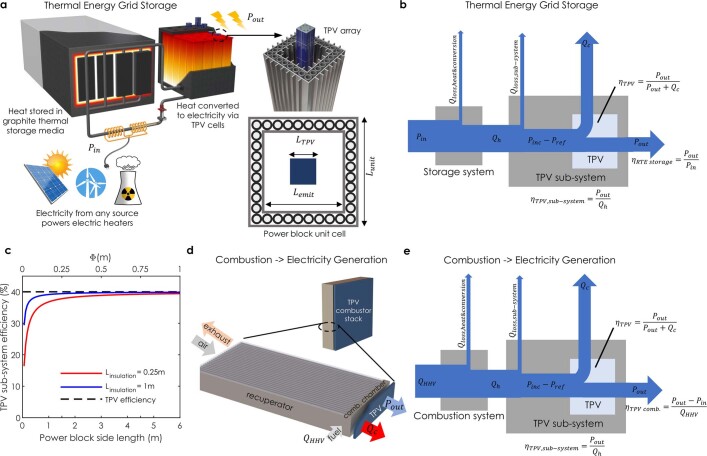
TPV applications. a) Conceptual illustration of TEGS^1^, which takes in electricity, converts it to heat via Joule heating, stores the heat in insulated graphite blocks, and then uses TPV for conversion of heat to electricity. A unit cell of the power block is also shown. B) Sankey diagram showing the energy flows in the TEGS system at scale and different efficiency metrics. c) The relationship between TPV sub-system efficiency and power block size or volume to surface area ratio, Φ, assuming the system is a cube. d) Conceptual illustration of a combustion-based electricity generation system using TPV. The system consists of an all-ceramic recuperator, similar to a printed circuit heat exchanger, with the end comprising of a combustion chamber. Air is preheated by exhaust and then combined with fuel for combustion near the end facing the TPV. The hot exhaust then delivers heat to the ceramic which radiates it to the TPV. e) Sankey diagram showing energy flows in a combustion-based TPV system at scale.

The cells are 1.4/1.2 eV and 1.2/1.0 eV tandem devices optimized for the 1,900–2,400 °C emitter temperature range (Fig. 1) for the thermal energy grid storage (TEGS) application^1,17^. TEGS is a low-cost, grid-scale energy storage technology that uses TPVs to convert heat to electricity above 2,000 °C, which is a regime inaccessible to turbines. It is a battery that takes in electricity, converts it to high-temperature heat, stores the heat and then converts it back to electricity by TPVs on demand. Although TEGS was initially conceived with a molten silicon storage medium^18^, a graphite storage medium is even lower cost (US$0.5 per kg), and the projected capital cost per unit energy (CPE) is less than US$10 per kWh (ref. ^19^). This cost is so low, it would enable TEGS to meet the proposed cost targets (<US$20 per kWh) for long-duration energy storage that would allow renewable energy with storage to be cost-competitive with fossil fuels^20–22^. As a result, the proliferation of TEGS could ultimately enable abatement of approximately 40% of global CO_2_ emissions, by decarbonizing the electricity grid (approximately 25% of emissions) and then enabling CO_2_-free electricity to charge vehicles in the transportation sector (approximately 15% of emissions)^23^. Reaching a TPV efficiency of 40% is notable, because it means that TEGS, as well as a range of other potential applications, are now feasible. These applications include other energy storage technologies^2^, natural gas, propane or hydrogen-fuelled power generation^3–9^, and high-temperature industrial waste heat recovery (Methods and Extended Data Fig. 1).

## High-efficiency TPV cells

The efficiency of a TPV cell is defined differently from that of a solar cell because, unlike a solar cell, a TPV system can preserve and later convert the energy in sub-bandgap photons. This is because, in the contexts in which TPV is envisioned to be used, the TPV cell has a high view factor to the emitter. This means that sub-bandgap photons can be reflected back to the emitter by the TPV cell (Fig. 1b), which is different from a solar cell and the sun. By reflecting unconverted photons, the energy of the sub-bandgap light is preserved through reabsorption by the emitter. The reflected and subsequently reabsorbed light helps to keep the emitter hot, thereby minimizing the energy input required to heat the emitter. As a result, the efficiency of a TPV cell is given by

1 $${\eta }_{{\rm{TPV}}}=\frac{{P}_{{\rm{out}}}}{{P}_{{\rm{out}}}+{Q}_{{\rm{c}}}}=\frac{{P}_{{\rm{out}}}}{{P}_{{\rm{inc}}}-{P}_{{\rm{ref}}}}$$

In equation (1),$\,{P}_{{\rm{out}}}\,$is the electric power generated by the TPV cell (that is, ${P}_{{\rm{out}}}={V}_{{\rm{oc}}}{I}_{{\rm{sc}}}{\rm{FF}}$), where ${V}_{{\rm{oc}}}$ is the open circuit voltage, ${I}_{{\rm{sc}}}$ is the short-circuit current and ${\rm{FF}}$ is the fill factor of the current–voltage (IV) curve. The total heat absorbed and generated in the cell is denoted by ${Q}_{{\rm{c}}}$, which is made up of the heat generated by parasitic absorption in the semiconductor or metal reflector, thermalization losses due to excess incident photon energy, Joule heating losses due to current flow and non-radiative recombination losses. The net energy received by the cell is equivalent to ${P}_{{\rm{out}}}+{Q}_{{\rm{c}}}$ and can also be expressed as ${P}_{{\rm{inc}}}-{P}_{{\rm{ref}}}$, where ${P}_{{\rm{inc}}}$ is the incident energy and ${P}_{{\rm{ref}}}$ is the reflected energy. Based on equation (1), to increase TPV efficiency, one must increase the power output ${P}_{{\rm{out}}}$ and/or reduce the amount of heat absorbed and generated in the cell (${Q}_{{\rm{c}}}$). The efficiency, ${\eta }_{{\rm{TPV}}}$, is the metric we use here because it is the conventional and generalizable metric used to describe the performance of a cell–emitter pair independent of other system-level characteristics^12^. The efficiency of a full system involving TPVs may be less than ${\eta }_{{\rm{TPV}}}$ due to system-specific losses. However, these system-level losses can become negligible in the case of TEGS or a large-scale combustion-based electricity generation system^1,24^ (Methods and Extended Data Fig. 1).

The high emitter temperatures targeted here for TEGS and other applications allow higher bandgap cells of at least 1.0 eV to be used instead of the low-bandgap, InGaAs- or GaSb-based cells traditionally used for TPV. This is key, because the spectrum of light redshifts towards longer wavelengths as the radiator temperature is lowered, which is why traditional TPV cells that are paired with emitters of less than 1,300 °C are typically based on 0.74 eV InGaAs or 0.73 eV GaSb. Considerable work on low-bandgap semiconductors has been undertaken with the envisioned application of converting heat from natural gas combustion^3–9^, concentrated solar power^24^, space power applications^25,26^ and, more recently, energy storage^1,2,27^. This pioneering body of work has led to the identification of three key features that now enable TPVs to become a competitive option for converting heat to electricity commercially: high-bandgap materials paired with high emitter temperatures, high-performance multi-junction architectures with bandgap tunability enabled by high-quality metamorphic epitaxy^16^ and the integration of a high-reflectivity BSR for band-edge filtering^11,13^.

With respect to higher bandgaps, they increase efficiency because there is an almost constant penalty on voltage of around 0.3–0.4 V, due to the thermodynamic requirements on the radiative recombination rate^28^. As a result, this unavoidable loss penalizes lower bandgap cells more than higher bandgap cells, because this loss makes up a smaller fraction of the voltage for higher bandgap materials. Using higher bandgap materials also needs to be accompanied by operation at higher temperatures to maintain sufficiently high power density, which scales with the emitter temperature to the fourth power. Operation at high power density is critical for TPV economics because the cell costs scale with their area, and if the power generation per unit area increases, the corresponding cost per unit power (CPP) decreases^29^.

With respect to BSRs, a highly reflective BSR is critical to minimize ${Q}_{{\rm{c}}}$. Highly reflective BSRs provide the additional benefit of boosting open-circuit voltage, because they also improve recycling of luminescent photons generated by radiative recombination^30–32^. This effect has led to regular integration of BSRs with solar PV cells, which provides a template for their use in TPVs. With these important lessons from previous work in mind, the cells developed here are 1.2/1.0 eV and 1.4/1.2 eV two-junction designs intended for the TEGS application with emitter temperatures between 1,900 and 2,400 °C (ref. ^1^). Multi-junction cells increase efficiency over single junctions by reducing hot carrier thermalization losses and reducing resistive losses by operating at a lower current density. The cells were based on the inverted metamorphic multi-junction architecture pioneered at the National Renewable Energy Laboratory (NREL)^33–35^.

**Extended Data Fig. 2:**
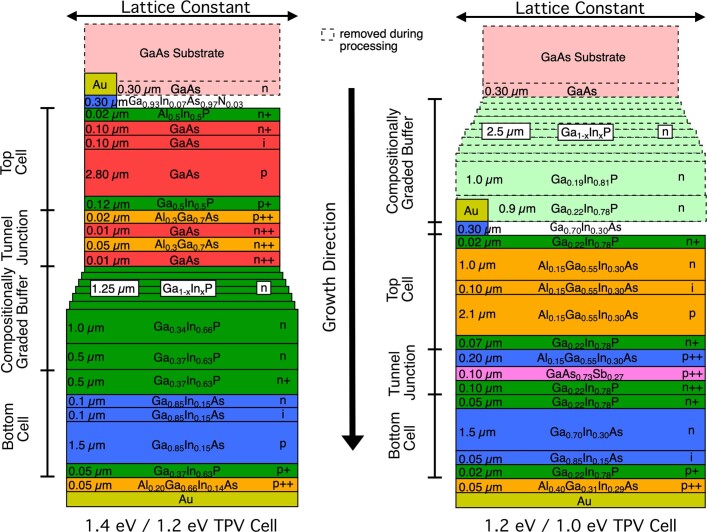
Tandem device structures. Device structures of the 1.4/1.2-eV and the 1.2/1.0-eV tandems.

The first cell design uses lattice-mismatched 1.2 eV AlGaInAs and 1.0 eV GaInAs top and bottom junctions, where the lattice mismatch is with respect to the crystallographic lattice constant of the GaAs substrate on which they are grown. The second design uses a lattice-matched 1.4 eV GaAs top cell and a lattice-mismatched 1.2 eV GaInAs bottom cell, taking advantage of the inherently higher material quality of lattice-matched epitaxy in the GaAs cell (Fig. 1c, Fig. 1d and Extended Data Fig. 2). The lower bandgap 1.2/1.0 eV tandem offers the potential for higher power density than the 1.4/1.2 eV tandem because it converts a broader band of the incident spectrum, and consequently the requirements on the BSR are less stringent to obtain high efficiency^27^. Higher power density can also be a practical engineering advantage. On the other hand, although the 1.4/1.2 eV tandem has a lower power output, the reduced current density of this bandgap combination potentially enables higher efficiency than the 1.2/1.0 eV tandem if resistive losses are an issue.

## TPV efficiency measurement results

**Fig. 2:**
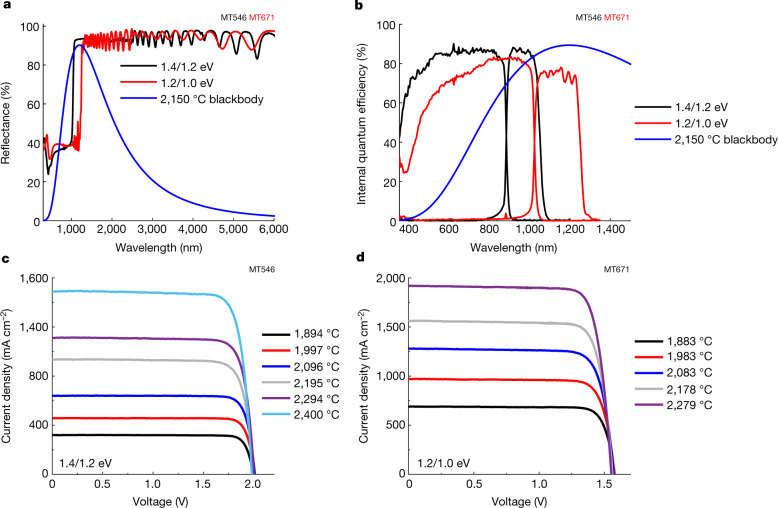
TPV characterization. **a**, Reflectance of the 1.4/1.2 eV and 1.2/1.0 eV tandems. The 2,150 °C blackbody spectrum is shown for reference, which is the average emitter temperature in the TEGS application. **b**, Internal quantum efficiency (IQE) of the 1.4/1.2 eV and 1.2/1.0 eV tandems. The EQE is shown in Extended Data Fig. 3. **c**, **d**, Current density–voltage curves measured in the efficiency setup at varying emitter temperatures for the 1.4/1.2 eV (**c**) and 1.2/1.0 eV (**d**) tandems.

**Fig. 3:**
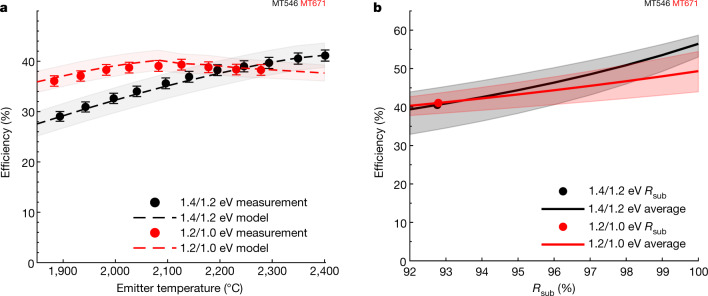
TPV efficiency. **a**, TPV efficiency measured at different emitter temperatures ranging from approximately 1,900 °C to 2,400 °C. The error bars indicate the uncertainty of the efficiency measurement, which is discussed in Methods. The dashed lines show the model predictions and the shaded regions show the uncertainty in the model predictions (see Methods). **b**, Predicted efficiency of the 1.4/1.2 eV and 1.2/1.0 eV tandems as the weighted sub-bandgap reflectance (${R}_{{\rm{sub}}}$) is extrapolated assuming a W emitter with AR = 1 and VF = 1 and a 25 °C cell temperature (Extended Data Fig. 5). The solid lines show the average efficiency within the TEGS operating temperature range of 1,900 °C to 2,400 °C. The shaded bands show the maximum and minimum efficiencies within the temperature range. The dots show the present value of ${R}_{{\rm{sub}}}$ based on the measured reflectance in Fig. 2a weighted by the W AR = 1, VF = 1 spectrum.

The TPV cell fabrication, measurement and modelling details are provided in the Methods. We refer to the two tandems by their bandgaps: 1.4/1.2 eV and 1.2/1.0 eV. Reflectance measurements are shown in Fig. 2a and internal quantum efficiency is given in Fig. 2b. The sub-bandgap spectral weighted reflectance for the 2,150 °C blackbody spectrum is 93.0% for the 1.4/1.2 eV tandem and 93.1% for the 1.2/1.0 eV tandem. The 2,150 °C blackbody spectrum shape is shown throughout for reference, because 2,150 °C is the average emitter temperature in the TEGS application and in the measurements. See Extended Data Figs. 4 and 5a for the measured spectrum and a comparison between the blackbody spectrum shape and the spectrum under which the cells were characterized. Current density versus voltage measurements were performed under a tungsten halogen bulb emitter and results for a range of emitter temperatures relevant to the TEGS application (approximately 1,900–2,400 °C) are shown in Fig. 2c, 2d. As expected, the 1.2/1.0 eV tandem had lower voltage but higher current density than the 1.4/1.2 eV tandem. The non-monotonic change in ${V}_{{\rm{oc}}}$ at the highest emitter temperatures was because of increasing cell temperature (Extended Data Fig. 6a) due to the presence of a heat flux sensor (HFS) used for the efficiency measurement, that undesirably also impeded heat flow. Figure 3a shows the efficiency measurement at the same range of emitter temperatures, which was accomplished by simultaneously measuring ${Q}_{{\rm{c}}}$ and ${P}_{{\rm{out}}}$.

The results for the 1.4/1.2 eV tandem showed increasing efficiency with increasing emitter temperature, and the efficiency exceeded 40% at 2,350 °C, which is within the target range of 1,900–2,400 °C needed for the TEGS application. At 2,400 °C, the efficiency was as high as 41.1 ± 1%, whereas the average efficiency between 1,900 and 2,400 °C was 36.2%. The electrical power density was 2.39 W cm^–2^ at the maximum emitter temperature of 2,400 °C. The rate of increase of efficiency with temperature slowed at high emitter temperatures due to a reduction in FF, because of increasing series resistance losses and the diminishing increase in ${J}_{{\rm{sc}}}$ due to the cell becoming current-limited by the bottom cell at approximately 2,250 °C.

The results for the 1.2/1.0 eV tandem showed greater efficiency than for the 1.4/1.2 eV tandem at lower emitter temperatures because of its lower bandgaps. The efficiency of the 1.2/1.0 eV tandem reached a maximum of 39.3 ± 1% at 2,127 °C, quite close to 2,150 °C, which is the temperature at which our device model predicted this bandgap combination would be optimal^27^. The average efficiency between 1,900 and 2,300 °C was 38.2% and the efficiency remained high across a 400 °C range of emitter temperatures. This is particularly worth noting for the TEGS application because it indicates consistently high efficiency can be achieved even as the emitter temperature varies during the discharging process of the TEGS system. The reduction in efficiency beyond this temperature was due to the increasing series resistance losses and the diminishing increase in ${J}_{{\rm{sc}}}$ due to the cell becoming current-limited by the bottom cell at temperatures greater than 2,150 °C. The electrical power density was 2.42 W cm^–2^ at the maximum emitter temperature measured of 2,279 °C, and it was 1.81 W cm^–2^ at the maximum efficiency point at the emitter temperature of 2,127 °C. Comparing the performance of the two cells across the range of emitter temperatures, they exhibit different characteristics that are advantageous for TEGS. The efficiency of the 1.2/1.0 eV tandem is less sensitive to changes in emitter temperature, has a higher electrical power density at a given emitter temperature and has a higher efficiency averaged over the emitter temperatures. However, the 1.4/1.2 eV tandem can reach higher efficiency at the highest emitter temperatures.

Figure 3a also shows model predictions for efficiency and the corresponding uncertainty of the model prediction. The good agreement obtained between the modelled and measured performance supports and validates the accuracy of the efficiency measurement and of the calorimetry-based method used to measure efficiency. In addition, the good agreement indicates that the model can be extended to extrapolate how the performance would change with additional improvements or at other operating conditions. The most important TPV cell property that could be improved is its spectral-weighted sub-bandgap reflectance, ${R}_{{\rm{sub}}}$. Figure 3b shows how the efficiency would change if ${R}_{{\rm{sub}}}$ could be increased. To extrapolate the results to a real TPV system, here we assume that the emitter is tungsten (W), as it is in the TEGS system, and that the area ratio between the emitter and cell is AR = 1, the view factor is ${\rm{VF}}=1$ and the cell temperature is 25 °C (Extended Data Fig. 5). In this prediction, for a 2,200 °C emitter temperature, the efficiency of the 1.4/1.2 eV tandem exceeds 50% at ${R}_{{\rm{sub}}}=97 \%$. The reason this is worth noting is because the present value of ${R}_{{\rm{sub}}}\,$is considerably lower than what was achieved with the air bridge approach recently demonstrated by Fan et al.^15^. Their work demonstrating a reflectivity of more than 98% charts a pathway towards further efficiency improvements. If the air bridge approach developed by Fan et al. could be combined with the advancements demonstrated here, it could lead to efficiencies greater than 56% at 2,250 °C, or greater than 51% averaged over the 1,900–2,400 °C temperature range.

## Conclusions

We report two-junction TPV cells with efficiencies of more than 40% using an emitter with a temperature between 1,900 and 2,400 °C. The efficiency of the 1.4/1.2 eV tandem reaches 41.1 ± 1% at 2,400 °C, with an average of 36.2% over the target temperature range. The efficiency of the 1.2/1.0 eV tandem reaches 39.3 ± 1% and varies very little over a wide temperature range with an average efficiency over the 1,900–2,300 °C temperature range of 38.2%. This high performance is enabled by the usage of multi-junction cells with bandgaps of at least 1.0 eV, which are higher bandgaps than have been traditionally used in TPVs. The higher bandgaps enable the use of higher emitter temperatures, which correspond to the temperature range of interest for the low-cost TEGS energy storage technology^1^. This temperature range is also applicable for natural gas or hydrogen combustion, and further demonstration of integrated systems is warranted.

Reaching 40% efficiency with TPVs is notable from the standpoint that it now renders TPV as a heat engine technology that can compete with turbines. An efficiency of 40% is already greater than the average turbine-based heat engine efficiency in the United States (Fig. 1a)^36–38^, but what could make TPVs even more attractive than a turbine is the potential for lower cost (CPP < US$0.25 per W)^1,24^, faster response times, lower maintenance, ease of integration with external heat sources and fuel flexibility. This is noteworthy  because turbine costs and performance have already reached full maturity, so there are limited prospects for future improvement, as they are at the end of their development curve. TPVs, on the other hand, are very early in their progress down a fundamentally different development curve. Consequently, TPVs have numerous prospects for both improved efficiency (for example, by improving reflectivity and lowering series resistance) and lowering cost (for example, by reusing substrates and cheaper feedstocks). Thus, the demonstration of 40% efficiency represents an important step towards realizing the potential that can be achieved with increased attention and funding in the coming years as commercial applications emerge and become profitable.

## Methods

### TPV applications

Turbines proliferated because of their high efficiency (25–60%) and their low CPP generated (US$0.5–1 per W). However, as turbines intrinsically require moving parts, there are corresponding requirements on the high-temperature mechanical properties of the materials of construction, as they are subject to centrifugal loads. Thus, they have reached their practical limits in terms of cost and efficiency, barring a materials discovery that would allow them to operate at substantially higher turbine inlet temperatures than the current values of approximately 1,500 °C for Brayton cycles and approximately 700 °C for Rankine cycles^29^. Solid-state heat engines such as TPVs, which have no moving parts, possess an advantage in this sense, enabling operation at significantly higher temperatures than turbines. TPVs can enable new approaches to energy storage^1,2^ and conversion^3–9^ that use higher temperature heat sources.

In this section, we highlight two promising applications for high-bandgap tandem TPVs paired with high-temperature heat sources: (1) TEGS^1^ and (2) combustion-driven electricity generation. We also discuss the importance of TPV efficiency in relation to the system-level efficiency metrics relevant to these applications.

TEGS, which is conceptually illustrated in Extended Data Fig. 1a, takes in electricity, converts it to heat by joule heating, stores the heat in a bank of large graphite blocks and then converts it back to electricity through TPVs. The heat is transferred to different parts of the system using mechanically pumped liquid metal tin^45^ and a graphite infrastructure, as demonstrated by Amy et al.^1,17,18^. The blocks store the heat and when electricity is desired, the liquid metal retrieves the heat and delivers it to a power block containing TPV cells that convert light emitted by the hot infrastructure. For a storage system, the primary efficiency metric is the round trip efficiency (RTE) described by the ratio of the output electrical power (${P}_{{\rm{out}}}$) to the input electrical power ${P}_{{\rm{in}}}$. For TEGS, ${P}_{{\rm{in}}}$ is primarily the electricity supplied to the resistance heaters, but also includes a contribution from pumping power requirements for the liquid tin heat transfer fluid and the heat exchanger for cell cooling. The Sankey diagram of the TEGS system is shown in Extended Data Fig. 1b.

For any system using TPVs, a subsystem efficiency can be defined as the ratio of the electric power output to the energy input to the emitter at steady state, ${Q}_{{\rm{h}}}$, such that ${\eta }_{{\rm{TPV}},{\rm{subsystem}}}={{P}_{{\rm{out}}}/Q}_{{\rm{h}}}$ (Fig. 1b and Extended Data Fig. 1b). ${\eta }_{{\rm{TPV}},{\rm{subsystem}}}\,$may be less than ${\eta }_{{\rm{TPV}}}$ due to view factor or convective losses from the emitter or cell, or other heat losses from the emitter to the environment (${Q}_{{\rm{loss}},{\rm{subsystem}}}$). Therefore, ${Q}_{{\rm{h}}}=\left({P}_{{\rm{out}}}/{\eta }_{{\rm{TPV}}}\right)+{Q}_{{\rm{loss}},{\rm{subsystem}}}$ and ${\eta }_{{\rm{TPV}},{\rm{subsystem}}}={\eta }_{{\rm{TPV}}}(1-\frac{{Q}_{{\rm{loss}},{\rm{subsystem}}}}{{Q}_{{\rm{h}}}}).$ Assuming no convective loss due to operation in a vacuum and negligible view-factor losses, then ${\eta }_{{\rm{TPV}},{\rm{subsystem}}}\approx {\eta }_{{\rm{TPV}}}$if ${Q}_{{\rm{loss}},{\rm{subsystem}}}$, which scales with the outer surface area of the power block, is small as compared with the energy conversion taking place inside the power block, which scales with its volume. This can be accomplished by increasing the scale of the system such that the heated material has a large volume to surface area ratio, *Φ*, and heat losses from the surfaces can be minimized with proper insulation^24^, and if the emitter surface and TPV module have a large surface area to perimeter ratio such that the view factor between them approaches one. This can be the case for TEGS or a large-scale combustion system, and it is a critically important aspect of achieving a high value for ${\eta }_{{\rm{TPV}},{\rm{subsystem}}}$ (refs. ^1,24^).

To illustrate the importance of *Φ*, Extended Data Fig. 1a shows a single unit cell of the TEGS power block, which is composed of a tungsten cavity emitter heated by pumped liquid tin, emitting to an array of TPV cells. The nominal dimensions of the TPV array, ${L}_{{\rm{TPV}}}$, and emitter, ${L}_{{\rm{emit}}}$, are 10 cm and 40 cm, respectively. The area ratio ${\rm{AR}}=\frac{{A}_{{\rm{emitter}}}}{{A}_{{\rm{TPV}}}}=4$ and the emitter material is tungsten based on previous optimization^1^. The graphite pipes, which carry the liquid tin heat transfer fluid and supply energy to the tungsten emitter surface, are 2 cm in diameter. Therefore, the side length of one unit cell of the power block is ${L}_{{\rm{unit}}}=44{\rm{cm}}.$ We note that although fins on the emitter can be used to increase the volumetric power density of the system, in this example we assume no fins are used for simplicity. In this example, we also assume that the depth dimensions of all components are equivalent, and that convective losses and view factor losses are negligible.

Heat losses from the exterior surface of the power block to the environment can be expressed as ${Q}_{{\rm{loss}},{\rm{subsystem}}}={hA}({T}_{{\rm{h}}}-{T}_{\infty })$, where $h$ is the overall heat transfer coefficient representing losses to the environment. The value of $h$ is dominated by conduction through the graphite insulation such that $h\approx k/{L}_{{\rm{insulation}}}$, where $k$ is the thermal conductivity of graphite insulation ($k\approx$1 W m^–1^ K^–1^ at 2,150 °C) and ${L}_{{\rm{insulation}}}$ is the insulation thickness. Although its thermal conductivity is moderate, graphite insulation is the only economical option for insulating systems above 1,700 °C (ref. ^46^). $A$ is the external surface area of the power block, ${T}_{{\rm{h}}}$ is the average temperature of the power block (2,150 °C) and ${T}_{\infty }$ is the temperature of the environment (25 °C).

Considering a single unit cell of the dimensions discussed above and using tungsten spectral properties and an emitter temperature ${T}_{{\rm{h}}}$ = 2,150 °C, our TPV model predicts ${P}_{{\rm{out}}}$=11.4 W per cm^2^ of cell area and ${\eta }_{{\rm{TPV}}}$= 40% for the 1.2/1.0 eV tandem. Considering the entire volume of the unit cell, this leads to a volumetric electric power density of 240 kW m^–3^. Assuming that the power block is a cube, Extended Data Fig. 1c shows ${\eta }_{{\rm{TPV}},{\rm{subsystem}}}$ as a function of the side length of the power block (excluding the insulation) as well as $\Phi$ for two different graphite insulation thicknesses. The results show that ${\eta }_{{\rm{TPV}},{\rm{subsystem}}}\,$approaches ${\eta }_{{\rm{TPV}}}\,$for power block length scales of approximately 1 m when the system is appropriately insulated. The results also indicate that TPVs are well-suited for large-scale systems, as it is challenging to achieve high system efficiencies with power block length scales of less than 1 m. In characterizing the RTE of TEGS (Extended Data Fig. 1b), other losses are due to the energy conversion of electricity to heat in the resistive heaters (<1%) and heat losses from the thermal storage media (approximately 1% per day), but they can be negligibly small^1^. Therefore, the RTE can be dominated by ${\eta }_{{\rm{TPV}}}$.

Here it is important to note that a RTE of 40–55% as is targeted in the TEGS application is low as compared to other options, such as Li-ion batteries, which have RTEs of more than 70%. However, several studies have pointed out that to enable full penetration of renewables onto the grid, a one to two order of magnitude decrease in CPE is required, owing to the need for long storage durations^20–22^. It is from this perspective that the RTE can be sacrificed, as long as it is above approximately 35% (ref. ^1^), provided it enables accession of much lower cost. Thus, technoeconomic analyses indicate that a technology with a tenfold lower CPE, yet a twofold lower efficiency as compared with Li-ion batteries, is still more economically attractive^1,20–22^.

Another promising application for TPVs is electricity generation in which the heat source is the combustion of fuel^3–9,47^. The temperature regime examined here is accessible by combustion of natural gas or hydrogen, which could be made into an efficient power generation system by using recuperators made from refractory metals and oxides^3,47^. Extended Data Figure 1d shows a modular combustion-driven TPV concept. Air enters a recuperator and is preheated by exchanging heat with the outgoing exhaust. The preheated air mixes with fuel, combusts and transfers heat to the emitter wall, which irradiates to the TPVs. Here, the important metric is the first-law thermal efficiency defining the ratio of net work output to the primary energy input (Extended Data Fig. 1e). The net work output is ${P}_{{\rm{out}}}-{P}_{{\rm{in}}}$, where ${P}_{{\rm{out}}}$ is the electric power output from the TPVs and ${P}_{{\rm{in}}}$ is the work input for pumping required for gas circulation and the TPV liquid cooling. The primary energy input is the higher heating value of the fuel, ${Q}_{{\rm{HHV}}}$. The combustor modules are stacked to create an array of length scale of around 1 m (Extended Data Fig. 1c), the side walls of each module are adiabatic by symmetry and the entire block of modules can be insulated at the outermost edges. A TPV panel that is close and opposite the emitter array has an area to perimeter ratio that is large and minimizes view-factor losses from the edges. Other heat losses can occur through the exhaust because of an imperfect recuperator. However, the efficiency at which the chemical energy in the fuel, ${Q}_{{\rm{HHV}}}$, is converted to ${Q}_{{\rm{h}}}$ for TPV systems (that is, ${Q}_{{\rm{h}}}/{Q}_{{\rm{HHV}}}$) can be approximately 90% (ref. ^3^).

These two examples (TEGS and combustion-driven electricity generation) illustrate the importance of $${\eta }_{{\rm{TPV}}}$$, which dominates system-level efficiencies for an appropriately designed system at scale. Assuming that the other losses can be made negligible, our work demonstrates a solid-state heat engine (terrestrial heat source) with an efficiency higher than the average heat engine efficiency in the United States, which is lower than 35% based on primary energy inputs and electricity output^38^. An efficiency of 40% is also higher than most steam cycles, and is in the same range as simple cycle gas turbines^48^. Thus, 40% represents a major step forward (Fig. 1a), as this is a type of heat engine that has the potential to compete with turbines by exhibiting comparable efficiency and potentially even lower CPP, for example less than $0.25 per W (refs. ^1,24^). To properly contextualize why this has broad-reaching implications, it should be appreciated that over the last century a range of alternative heat engines, such as thermoelectrics^49^, thermionics^50^, TPVs^12^, thermally regenerative electrochemical systems^51^, thermoacoustic engines^52^ and Stirling engines^53,54^, have been developed. All these technologies have some intrinsic advantage(s) over turbines, such as low maintenance, no moving parts and/or easier integration with an external heat source, yet none of them have previously been able to compete with the efficiency and CPP of turbines for large-scale heat to electricity conversion.

### TPV cell growth and processing details

Extended Data Figure 2 shows the device structures of the tandem cells. All materials were grown by atmospheric pressure organometallic vapour phase epitaxy using trimethylgallium, triethylgallium, trimethylindium, triethylaluminium, dimethylhydrazine, arsine and phosphine. Diethylzinc and carbon tetrachloride were used as p-type dopant sources and hydrogen selenide and dislane were used as n-type dopant sources. Growth took place in a purified hydrogen gas flow of 6 litres per minute. Substrates were n-type (100) GaAs with a 2° offcut towards the (111)B plane, and all devices were grown in an inverted configuration. For both types of cells, the substrate was prepared by first etching in NH_4_OH:H_2_O_2_:H_2_O (2:1:10 by volume). The substrate was then mounted on a graphite susceptor and heated inductively to 700 °C under an arsine overpressure, followed by an approximately 10 min deoxidization under arsine.

Growth of the 1.4/1.2 eV tandem started with a 0.2 µm GaAs buffer and was then followed by a 0.5 µm GaInP etch stop layer. Then, 0.1 µm of GaInAsN:Se and 0.2 µm of GaAs:Se were deposited as the front contact layer. The top cell was grown, starting with a 0.02 µm AlInP window layer, then a 0.1 GaAs:Se emitter, a 0.1 µm undoped GaAs layer, a 2.8 µm GaAs:Zn base layer and a 0.12 µm GaInP back surface field (BSF) layer. Next, an AlGaAs:C/GaAs:Se/AlGaAs:Si quantum well tunnel junction was grown, followed by a GaInP compositionally graded buffer (CGB). The CGB consisted of 0.25 µm GaInP steps spanning the compositional range Ga_0.51_In_0.49_P to Ga_0.34_In_0.66_P at a rate of 1% strain per µm, with the final layer being a 1.0 µm Ga_0.34_In_0.66_P strain overshoot layer. The bottom cell was grown, consisting of a 1.0 µm Ga_0.37_In_0.63_P window, a 0.1µm Ga_0.85_In_0.15_As:Se emitter, a 0.1µm Ga_0.85_In_0.15_As i-layer, a 1.5 µm Ga_0.85_In_0.15_As:Zn base and a 0.05 µm Ga_0.37_In_0.63_P:Zn BSF. Finally, a 0.05 µm Al_0.20_Ga_0.66_In_0.14_As:Zn++ back contact layer was grown.

For the 1.2/1.0 eV design^27^, a 0.2 µm GaAs buffer layer was grown first, then a GaInP CGB consisting of 0.25 µm GaInP steps, spanning the range Ga_0.51_In_0.49_P to Ga_0.19_In_0.81_P, with the final layers being a 1.0 µm Ga_0.19_In_0.81_P strain overshoot layer and a 0.9 µm Ga_0.22_In_0.78_P step back layer lattice matched to the in-plane lattice constant of the Ga_0.19_In_0.81_P. A 0.3 µm Ga_0.70_In_0.30_As:Se front contact layer was grown next, followed by the top cell, starting with a 0.02 µm Ga_0.22_In_0.78_P:Se window, a 1.0 µm Al_0.15_Ga_0.55_In_0.30_As:Se emitter, an undoped 0.1 µm Al_0.15_Ga_0.55_In_0.30_As i-layer, a 2.1 µm Al_0.15_Ga_0.55_In_0.30_As:Zn base and a 0.07 µm Ga_0.22_In_0.78_P:Zn BSF. Then the tunnel junction, comprising a 0.2 µm Al_0.15_Ga_0.55_In_0.30_As:Zn layer, a 0.05 µm GaAs_0.72_Sb_0.28_:C++ layer and a 0.1 µm Ga_0.22_In_0.78_P:Se++ layer, was grown. Finally, the bottom cell was grown, comprising a 0.05 µm Ga_0.22_In_0.78_P:Se window, a 1.5 µm Ga_0.70_In_0.30_As:Se emitter, a 0.1 µm Ga_0.70_In_0.30_As:Zn i-layer and a 0.02 µm Ga_0.22_In_0.78_P:Zn BSF. Finally, a 0.05 µm Al_0.4_Ga_0.30_In_0.30_As:Zn++ back contact layer was grown.

After growth, an approximately 2-µm-thick reflective gold back contact was electroplated to the exposed back contact layer (the last semiconductor layer grown). The samples were bonded with low viscosity epoxy to a silicon handle and the substrates were etched away in NH_4_OH:H_2_O_2_ (1:3 by volume). Gold front grids were electroplated to the front surfaces through a positive photoresist mask, using a thin layer of electroplated nickel as an adhesion layer. The grids were nominally 10 µm wide, 100 µm apart and at least 5 µm thick. The samples were then isolated into individual devices using standard wet-chemical etchants and cleaved into single cell chips for characterization. The completed cells had mesa areas of 0.8075 cm^2^, with illuminated areas (discounting the single busbar but including the grid fingers) of 0.7145 cm^2^.

### Efficiency measurement

To measure the TPV cell efficiency, we seek direct measurement of the two contributing quantities in equation (1), the power output ${P}_{{\rm{out}}}={V}_{{\rm{oc}}}{I}_{{\rm{sc}}}{\rm{FF}}\,$ and the heat generated in the cell, ${Q}_{{\rm{c}}}$. To test the cells under a well-controlled and relevant spectrum (emission from tungsten between 1,900 and 2,400 °C for TEGS), a tungsten halogen lamp was used in combination with a concentrator. The concentrator consisted of a silver-plated elliptical reflector behind the lamp and a compound parabolic reflector (CPC) obtained from Optiforms that further concentrated the light onto the cell. At the base of the CPC, a water-cooled aluminium aperture plate was suspended above the TPV cell (Extended Data Fig. 7). The area of the aperture was 0.312 cm^2^ and the active area of the cell was 0.7145 cm^2^.

To keep the TPV cell cool it was mounted on a microchannel copper heat sink (M2, Mikros) that was water-cooled. To measure ${Q}_{{\rm{abs}}}$, a HFS, model gSKIN XP obtained from greenTEG, was placed between the cell and the heat sink. Thermally conductive adhesive tape held the HFS in place on the heat sink, and thermal paste provided thermal contact between the cell and the HFS. Electrical contact to the cell bus bars was accomplished using a pair of copper clips, which were both electrically and thermally isolated from the heat sink using a piece of insulation. A pair of wires was connected to the bottom of each copper clip to perform a four-wire measurement. The bottom side of the aluminium aperture plate was shielded with several layers of copper-coated Kapton and aluminium tape acting as a radiation shield to reduce the radiative transfer between the aperture plate and the TPV cell.

A d.c. power supply (Magna-Power) provided power to the tungsten halogen lamp and the voltage was controlled to achieve the desired emitter temperature. The lamp was rated for 5 kW at 3,200 K, but the temperature and power were tuned down to the desired emitter temperature by controlling the voltage to the lamp using the power supply. The emitter temperature was determined by measuring the resistance of the tungsten heating element in the lamp and using published correlations on the temperature dependence of the electrical resistivity and resistance of tungsten filaments in incandescent lamps^55^. First, the cold resistance of the bulb was measured at the point of the bulb junction and at the point of contact with the power supply to determine the resistance of the electrical leads to the bulb. The hot bulb resistance was measured by subtracting the electrical lead resistance from the total resistance as determined from the voltage and current input to the d.c. power supply. The heat sink was mounted onto the z-stage to allow for repeatable control of the TPV cell positioning with respect to the aperture, reflectors and lamp.

The TPV efficiency was measured by taking simultaneous measurements of ${P}_{{\rm{out}}}$ and ${Q}_{{\rm{c}}}$. The electric power was measured using a source meter (Keithley 2430) by sourcing the voltage and measuring the current density at the maximum power point, and ${Q}_{{\rm{c}}}$ was measured using the HFS beneath the cell. Owing to the temperature-dependent sensitivity of the HFS, the average HFS temperature, ${T}_{{\rm{s}}}$, was needed, which is taken from the average of the hot- and cold-side temperatures. The hot-side temperature was measured by a thermocouple placed underneath the cell. The cold-side temperature was determined iteratively using the thermal resistance of the sensor (4.167 K W^–1^), the measured heat flux and the cell temperature. From the calibration certificate from the manufacturer, the sensitivity $S(\mu {\rm{V}}\,{{\rm{W}}}^{-1}\,{{\rm{m}}}^{-2})$ is given by *S* = (*T*_s_ – 22.5)0.025 + 19.98.

### Emitter spectrum

The spectrum of the light source was measured using spectrometers in the visible (Ocean Insight FLAME) and in the near-infrared (NIR) (Ocean Insight NIRQUEST). The spectrometers were calibrated using a 1,000 W, 3,200 K quartz tungsten halogen bulb with known spectrum (Newport). Spectrum measurements at several temperatures can be found in Extended Data Fig. 4. To extrapolate the measured spectrum to a broader wavelength range, the spectrum was modelled by considering the literature values of the emission of tungsten^56^, the filament material, and transmission of quartz, for the envelope surrounding the bulb. Quartz transmission was calculated for a 3-mm-thick piece of quartz using optical constants from the literature^57^. The filament consists of tungsten coils with non-zero view factor to themselves. The coil geometry acts to smooth the spectral emission because light emitted by the inside of the coil has a high view factor to itself. Therefore, a geometric factor accounting for this smoothing was used as a fitting parameter to model the spectrum to extend it beyond the spectrometer measurement range. Extended Data Figure 5a shows a comparison between the spectrum described by the emission of tungsten with AR = 1 and VF = 1, a blackbody spectrum shape and the model, which was found to agree well with the measured spectrum. Owing to the good agreement, the modelled spectrum was then used to form the efficiency predictions. We refer to this spectrum as ${E}_{{\rm{TPV}}}\left(\lambda ,T\right)$ in the subsequent sections, where *λ* is wavelength.

Extended Data Figure 5b shows a comparison between the TPV model results under the lightbulb spectra with spectra corresponding to emitter/cell pairs with ${\rm{VF}}=1$, which allows the reflected light to be recycled (an example of these systems is shown in Extended Data Fig. 1). Modelling is shown for a tungsten emitter operating with ${\rm{AR}}=1$ and ${\rm{VF}}=1$, and for a blackbody emitter with ${\rm{VF}}=1$. The results show that the lightbulb spectra provide a characterization of TPV efficiency that is relevant to various higher intensity spectra experienced in TPV systems.

### Effective view factor

To compare the measured TPV cell performance to model predictions, the effective view factor, ${{\rm{VF}}}_{{\rm{eff}}},$ was deduced from *J*_sc_ which was computed from Osterwald^58^ and is shown in equations (2) and (3). We used an NREL-fabricated GaAs cell with measured EQE and a ${J}_{{\rm{sc}}}$ that was measured at NREL on an XT-10 solar simulator (AM1.5D, 1,000 W m^–2^) using a secondary calibration reference cell to set the intensity. Before an efficiency measurement, the GaAs cell was placed in the setup at the same location as the multi-junction cell using the z-stage. In equation (2), ${J}_{{\rm{sc}}}^{{\rm{TPV}}}$ is the short-circuit current of the GaAs cell measured in the efficiency setup, ${J}_{{\rm{sc}}}^{{\rm{G}}173{\rm{d}}}$ is the short-circuit current of the cell measured using the XT-10 simulator at NREL, ${E}_{{\rm{TPV}}}\left(\lambda ,T\right)$ is the spectral emissive power under the measured spectrum in the efficiency setup (Extended Data Fig. 4) and ${E}_{{\rm{G}}173{\rm{d}}}\left(\lambda \right)$ is the AM1.5D spectrum. Both spectra are in units of W m^–2^ nm^–1^. We define ${{\rm{VF}}}_{{\rm{eff}}}$ as the ratio of the actual irradiance in the efficiency setup, ${E}_{{\rm{irradiance}}}^{{\rm{TPV}}}$, to the full irradiance for the spectral emissive power at the same test temperature, $\int {E}_{{\rm{TPV}}}\left(\lambda ,T\right){\rm{d}}\lambda$ (equation (3)). The Emitter Spectrum section above discusses how ${E}_{{\rm{TPV}}}\left(\lambda ,T\right)$ was determined. Measurements of ${J}_{{\rm{sc}}}^{{\rm{TPV}}}$ were averaged across the range of emitter temperatures.

2 $${J}_{{\rm{s}}{\rm{c}}}^{{\rm{T}}{\rm{P}}{\rm{V}}}={{\rm{V}}{\rm{F}}}_{{\rm{e}}{\rm{f}}{\rm{f}}}\times \frac{{J}_{{\rm{s}}{\rm{c}}}^{{\rm{G}}173{\rm{d}}}}{1,\,000\,{\rm{W}}\,{{\rm{m}}}^{-2}}\times \frac{\int {E}_{{\rm{T}}{\rm{P}}{\rm{V}}}(\lambda ,T){\rm{E}}{\rm{Q}}{\rm{E}}(\lambda )\lambda {\rm{d}}\lambda }{\int {E}_{{\rm{G}}173{\rm{d}}}(\lambda ){\rm{E}}{\rm{Q}}{\rm{E}}(\lambda )\lambda {\rm{d}}\lambda }\times \int {E}_{{\rm{G}}173{\rm{d}}}(\lambda ){\rm{d}}\lambda $$

3 $${{\rm{VF}}}_{{\rm{eff}}}=\frac{{E}_{{\rm{irradiance}}}^{{\rm{TPV}}}}{\int {E}_{{\rm{TPV}}}\left(\lambda ,T\right){\rm{d}}\lambda }$$

${{\rm{VF}}}_{{\rm{eff}}}$ was then used to form the efficiency model predictions. A useful metric to enable comparisons with other systems is to define an effective view factor in relation to the blackbody spectrum. Equation (4) compares the TPV irradiance in our efficiency setup with that of the Planck distribution blackbody spectrum at the same test temperature.

4 $${{\rm{VF}}}_{{\rm{eff}},{\rm{black}}}=\frac{{E}_{{\rm{irradiance}}}^{{\rm{TPV}}}}{\int {E}_{{\rm{B}}}\left(\lambda ,T\right){\rm{d}}\lambda }$$

Because the shape of ${E}_{{\rm{TPV}}}\left(\lambda ,T\right)$ varies slightly with temperature, ${{\rm{VF}}}_{{\rm{eff}},{\rm{black}}}$ also changes slightly with temperature. Averaged across the emitter temperatures, for the 1.4/1.2 eV tandem ${{\rm{VF}}}_{{\rm{eff}},{\rm{black}}}=10.07 \%$ and for the 1.2/1.0 eV tandem ${{\rm{VF}}}_{{\rm{eff}},{\rm{black}}}=10.65 \%$. The differences are due to slight adjustments made to the setup between measurements of the two multi-junction cells.

### Efficiency validation

Equation (1) for TPV efficiency can also be written in terms of equation (5), where ${P}_{{\rm{inc}}}$ is the irradiance incident on the cell, ${P}_{{\rm{ref}}}$ is the flux reflected by the cell, ${P}_{{\rm{inc}},{\rm{a}}}$ is the above-bandgap irradiance, ${P}_{{\rm{inc}},{\rm{sub}}}$ is the sub-bandgap irradiance, ${R}_{{\rm{a}}}$ is the spectral-weighted above-bandgap reflectance and ${R}_{{\rm{sub}}}$ is the spectral-weighted sub-bandgap reflectance^27^. The denominator of the efficiency expression represents the net flux to the cell.

5 $${\eta }_{{\rm{T}}{\rm{P}}{\rm{V}}}=\frac{{P}_{{\rm{o}}{\rm{u}}{\rm{t}}}}{{P}_{{\rm{o}}{\rm{u}}{\rm{t}}}+{Q}_{{\rm{c}}}}=\frac{{V}_{{\rm{o}}{\rm{c}}}\,{J}_{{\rm{s}}{\rm{c}}}{\rm{F}}{\rm{F}}}{{P}_{{\rm{i}}{\rm{n}}{\rm{c}}}-{P}_{{\rm{r}}{\rm{e}}{\rm{f}}}}=\frac{{V}_{{\rm{o}}{\rm{c}}}\,{J}_{{\rm{s}}{\rm{c}}}{\rm{F}}{\rm{F}}}{{P}_{{\rm{i}}{\rm{n}}{\rm{c}}}-{P}_{{\rm{i}}{\rm{n}}{\rm{c}},{\rm{a}}}\hspace{0.25pt}{R}_{{\rm{a}}}-{P}_{{\rm{i}}{\rm{n}}{\rm{c}},{\rm{s}}{\rm{u}}{\rm{b}}}\hspace{0.25pt}{R}_{{\rm{s}}{\rm{u}}{\rm{b}}}}$$

The measured ${V}_{{\rm{oc}}}$, ${J}_{{\rm{sc}}}$ and ${\rm{FF}}$ are shown in Extended Data Fig. 8 and Extended Data Tables 1 and 2. To model the numerator or electric power portion of the efficiency expression (Extended Data Fig. 8), we used a well-established analytical model that takes values extracted from experiments as input parameters^59^. Using a flash simulator with known spectral irradiance, we first measured the cell performance under carefully controlled conditions of known spectrum with the cell temperature fixed at 25 °C. Using the model, we fit the data satisfactorily over an irradiance range of several orders of magnitude (shown for the 1.2/1.0 eV tandem in Extended Data Fig. 9a). The fitting was done using only three parameters: the geometric averaged dark current for the two junctions in the form of ${W}_{{\rm{o}}{\rm{c}}}=\frac{{E}_{{\rm{g}}}}{e}-{{\rm{V}}}_{{\rm{o}}{\rm{c}}}$ (ref. ^60^) where *E*_g_ is the bandgap and *W*_oc_ is the bandgap-voltage offset, the *n* = 2 component of the dark current and the effective lumped series resistance ${R}_{{\rm{series}}}$. We refer to these as the cell characteristic parameters.

**Extended Data Fig. 3:**
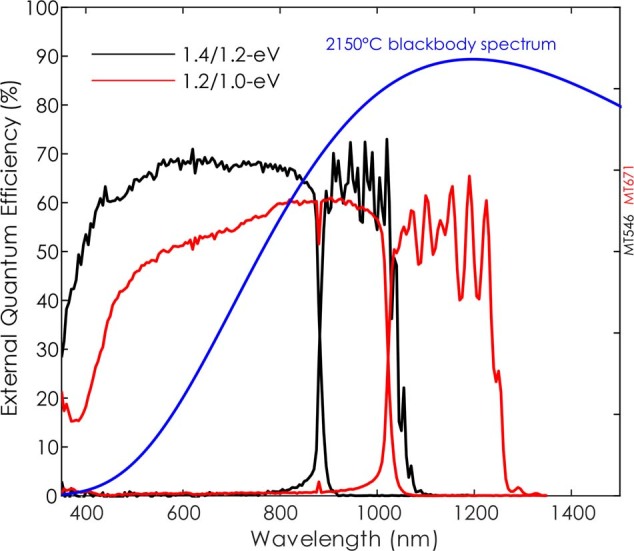
External Quantum Efficiency. The external quantum efficiency (EQE) of the two cells. The blue curve shows the 2150 °C blackbody spectrum for reference.

We then measured the IV performance parameters (${J}_{{\rm{sc}}},{V}_{{\rm{oc}}},{\rm{FF}}$) of the device as a function of the ratio of the top to bottom junction photocurrents under a continuous 1 sun simulator for which the spectral content can be varied. Using the measured EQE of the cells (Extended Data Fig. 3), the photocurrent ratio for a given emitter temperature can be calculated, and using reference cells^58^ the simulator was set to that photocurrent ratio for each emitter temperature. With the measured EQE and the cell characteristic parameters from above, we calculated the cell performance parameters and compared them to the measurements (shown for the 1.2/1.0 eV tandem in Extended Data Fig. 9b). The agreement supports the validity of the modelling process and its ability to correctly predict performance trends under a wide range of conditions—for both irradiance and emitter temperature (that is, spectrum).

**Extended Data Fig. 4:**
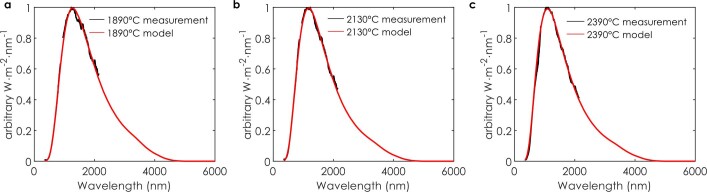
Emitter spectrum measurements and model. The emitter spectrum was measured at different emitter temperatures spanning the test temperature range. A model (Methods) was fit to the measurement and used to extend the spectra measurements to longer wavelengths. The spectral radiance goes to zero > ~4500 nm due to the presence of the quartz envelope around the bulb, as quartz is absorbing beyond this wavelength.

**Extended Data Fig. 5:**
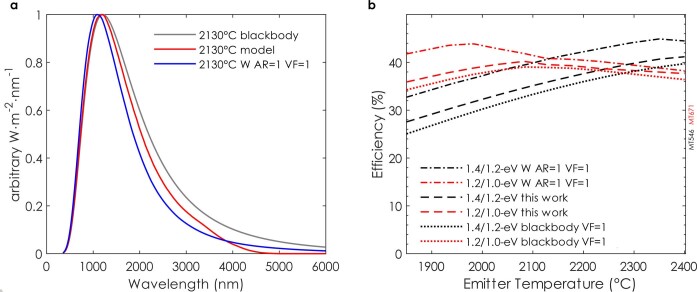
Comparison of spectra shapes. a) A comparison between the spectrum shape at an intermediate test temperature (2130 °C) The red curve shows the modeled spectrum which agrees well with the measurement (see Extended Data Fig. 4). The gray curve shows comparison to a blackbody spectrum shape at the same emitter temperature. The blue curve shows comparison to the spectrum described by the literature emission of tungsten with AR=1, VF=1. All curves are normalized by their peak to show the comparison in spectra shapes. The spectrum shape under which the cells were characterized (red curve) is similar to that of a blackbody (gray curve), particularly above bandgap. Comparison of modeled TPV efficiency under the spectrum in this work with emitters which could be incorporated into a TPV system in which the ${AR}$ and ${VF}$ allow for the reflected light to be recycled. Shown is a tungsten (W) emitter with ${AR}=1$ and ${VF}=1$ as well as a blackbody emitter (cavity) with ${VF}=1$. An example of systems which could have this geometry is shown in Extended Data Fig. 1. The W emitter results in a higher efficiency because the selective emissivity properties of W suppress some of the below-bandgap energy. Additionally, the W emitter causes the peak in efficiency to shift to lower temperature because the emissivity of W weights the spectrum towards shorter wavelengths. The blackbody emitter results in a lower efficiency because the high irradiance causes a larger penalty of series resistance loss due to the high current density. The comparison shows that the efficiency measured under the lightbulb spectrum in this work provides an appropriate and relevant characterization for TPV efficiency in a real TPV sub-system. In all cases, the cell temperature is 25 °C.

**Extended Data Fig. 6:**
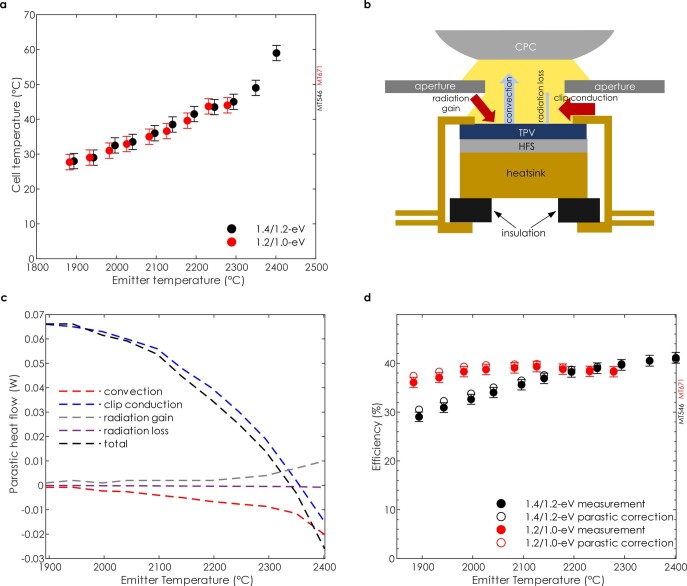
Cell temperature and parasitic heat flows. a) Cell temperature vs emitter temperature. The cell temperature increases with emitter temperature due to the heat flux sensor which undesirably impedes heat flow. b) Schematic (not to scale) showing parasitic heat flows in the experiment. c) Calculated parasitic heat flows for the 1.4/1.2-eV device. A positive value would act to increase the measured heat flow and reduce the measured efficiency, while a negative value would have the opposite effect. d) Comparison of the efficiency measurement (solid circles) to the measurement with the addition of the modeled parasitic heat flows (open circles) for both tandems.

**Extended Data Fig. 7:**
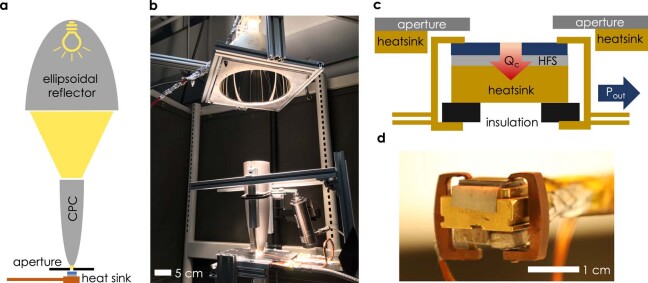
Experimental setup. a) Schematic of the concentrator setup showing the relative placement of the ellipsoidal and compound parabolic reflectors, water-cooled aperture, TPV cell, HFS, and heat sink. b) Image of the concentrator setup. c) Schematic of the heat and electricity flows through the measurement device. Electric power is extracted by two copper clips which interface with the cell bus bars on the top surface of the cell and are thermally and electrically insulated from the heat sink. d) Image of the cell on the heatsink with electrical leads. The aperture was removed for clarity.

**Extended Data Fig. 8:**
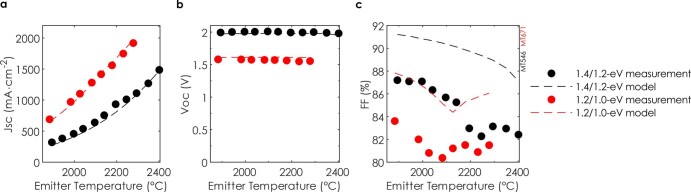
J_sc_, V_oc_, and FF. Modeled vs measured a) ${J}_{{sc}}$, b) ${V}_{{oc}}$, and c) ${FF}$. Good agreement can be seen between the measurement and model predictions. For each device, the ${FF}$ measurement and model exhibit the same trend and the minimum in ${FF}$ for 1.2/1.0-eV agrees well between the model and measurement which suggests good calibration of the emitter temperature.

**Extended Data Fig. 9:**
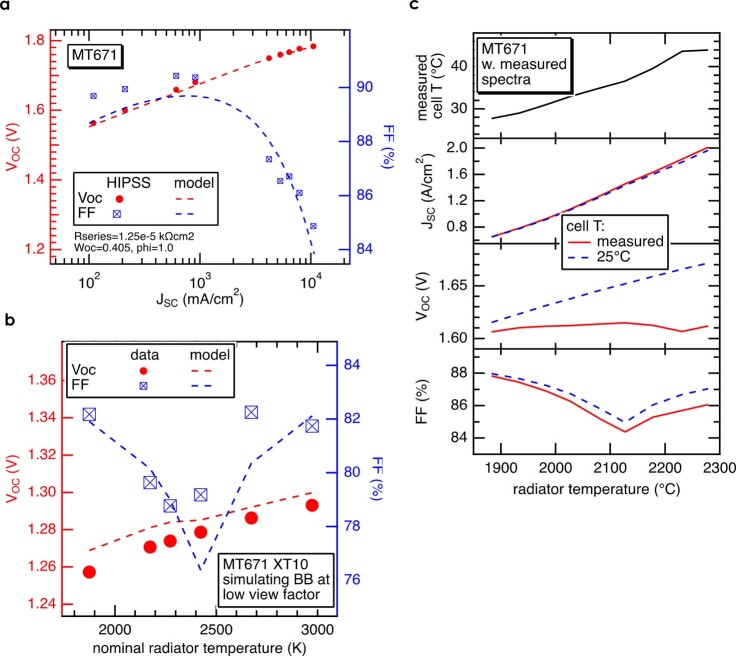
Electric power modeling. a) Measurements of ${V}_{{oc}}$ and ${FF}$ vs ${J}_{{sc}}$ for the 1.2/1.0-eV device under the high irradiance flash simulator over a wide range of irradiances, but fixed spectrum and fixed cell temperature at 25 °C. A model was fit to the data using the three fitting parameters to determine the cell characteristics. The measurement over a wide irradiance range is critical to extract the ${R}_{{series}}$ parameter under the high-irradiance conditions of interest. b) Low irradiance measurements of ${V}_{{oc}}$ and ${FF}$ under a continuous 1 sun simulator in which the spectral content could be varied to produce photocurrent ratios of the two junctions corresponding to different emitter temperatures. Cell temperature was fixed at 25 °C. The model was determined using the cell characteristic parameters which were extracted from fitting to the data over a wide range of irradiances. The good agreement suggests that the model can be used to predict ${V}_{{oc}}$, ${J}_{{sc}}$, ${FF}$ over a wide range of conditions (irradiance and spectra). c) Modeled cell performance parameters under the measured spectra showing a comparison between results for a 25 °C cell temperature and the measured cell temperature.

The measured spectra (Extended Data Fig. 4) were used along with the measured EQE to calculate the top and bottom junction photocurrents (equation (6)). With those as inputs to the model, and the cell characteristic parameters determined above, we computed the cell performance parameters under the actual efficiency measurement conditions. The cell temperature varies (Extended Data Fig. 6a). This was accounted for using a well-established model that works especially well for near-ideal devices, such as III–V devices. The model accounts for the temperature dependence through its effect on the intrinsic carrier density, and thus the dark current, and the effects of the bandgap variation with temperature^61,62^. Extended Data Figure 9c shows a comparison of the computed cell performance for a 25 °C cell and at the measured cell temperature for the 1.2/1.0 eV tandem.

6 $${J}_{{\rm{s}}{\rm{c}}}=\frac{q\times {{\rm{V}}{\rm{F}}}_{{\rm{e}}{\rm{f}}{\rm{f}}}}{c\times h}{\int }_{0}^{{\rm{\infty }}}{\rm{E}}{\rm{Q}}{\rm{E}}(\lambda ){E}_{{\rm{T}}{\rm{P}}{\rm{V}}}(\lambda ,T)\lambda {\rm{d}}\lambda $$

The spectral emissive power, ${E}_{{\rm{TPV}}}\left(\lambda ,T\right)$ was used to determine ${P}_{{\rm{inc}}}$ based on the emitter temperature, $T$, and ${{\rm{VF}}}_{{\rm{eff}}}$ (equation (7)). The reflectance, $\rho (\lambda )$, was measured on two different instruments owing to the range of the spectrum. The mid-infrared sub-bandgap reflectance was measured using a Fourier-transform infrared (FTIR) spectrometer (Nicolet iS50) with an integrating sphere accessory (PIKE Mid-IR IntegratIR). A copper aperture with area approximately 0.35 cm^2^ was used over the sample port, and the spot encompassed both the cell and the front grids. The above-bandgap and NIR sub-bandgap reflectance was measured using an ultraviolet-visible-NIR spectrophotometer (Cary 7000) with the diffuse reflectance accessory and with a spot size approximately 0.4 cm^2^ encompassing the cell and the front grids. ${P}_{{\rm{ref}}}$ was then calculated according to equation (8).

7 $${P}_{{\rm{inc}}}={{\rm{VF}}}_{{\rm{eff}}}{\int }_{0}^{{\rm{\infty }}}{E}_{{\rm{TPV}}}\left(\lambda ,T\right){\rm{d}}\lambda $$

8 $${P}_{{\rm{ref}}}={{\rm{VF}}}_{{\rm{eff}}}{\int }_{0}^{{\rm{\infty }}}{E}_{{\rm{TPV}}}\left(\lambda ,T\right)\rho (\lambda ){\rm{d}}\lambda $$

This approach to modelling the cells was used to predict the cell performance under the tungsten filament lighting conditions. The decomposition of reflectance into ${R}_{{\rm{a}}}$ and ${R}_{{\rm{sub}}}\,$portions (equation (4)) enabled the subsequent predictions of efficiency at higher ${R}_{{\rm{sub}}}$ shown in Fig. 3b.

### Heat transfer considerations

We examined the influence of different parasitic heat flows on the efficiency measurement. A schematic of the different parasitic heat flows is shown in Extended Data Fig. 6b and they are quantified in Extended Data Fig. 6c. Possible parasitic heat flows, ${Q}_{{\rm{parastic}}}$, are given by equation (9). A positive value of ${Q}_{{\rm{parastic}}}$ would act to increase the measured heat flow and reduce the measured efficiency, whereas a negative value of ${Q}_{{\rm{parastic}}}$ would have the opposite effect.

9 $${{Q}_{{\rm{parastic}}}=Q}_{{\rm{cond}},{\rm{clips}}}+{Q}_{{\rm{rad}},{\rm{gain}}}-{Q}_{{\rm{rad}},{\rm{loss}}}-{Q}_{{\rm{conv}},{\rm{loss}}}$$

For example, the aperture does not block all the light hitting the electrical leads. ${Q}_{{\rm{cond}},{\rm{clips}}}$ arises owing to conduction from the electric leads into the cell that is cooled by the heat sink, which by design are thermally stranded from the heat sink using insulation. To quantify this value, we performed measurements of the heat flow both with and without the electrical leads attached to the cell. In both cases the cell was operating at ${V}_{{\rm{oc}}}$ to avoid differences in heating due to power being extracted by the cell. The difference between the two heat flows is ${Q}_{{\rm{cond}},{\rm{clips}}}$. The results show that, at most emitter temperatures, the heat flow in the presence of the leads is larger than without, because the leads are thermally stranded while the cell is actively cooled. Thus, inclusion of such a term would lead to a higher efficiency than what is reported.

The next parasitic heat flow is due to radiation from the aperture plate to the cell, ${Q}_{{\rm{rad}},{\rm{gain}}}$. The temperature of the bottom of the aperture plate was measured with a thermocouple at the different emitter temperatures. Aperture temperatures varied from 43 °C at the lowest emitter temperature to 125 °C at the highest. The view factor between the aperture plate and the cell, ${F}_{{\rm{ac}}}$, was calculated from their geometry and spacing. The heat transfer from the aperture to the cell was calculated using a diffuse grey approximation according to equation 10, where *A*_ap_ is the area of the aperture plate and *A*_cell_ is the area of the cell.

10 $${Q}_{{\rm{rad}},{\rm{gain}}}=\frac{\sigma \left({T}_{{\rm{ap}}}^{4}-{T}_{{\rm{cell}}}^{4}\right)}{\frac{1-{\varepsilon }_{{\rm{cell}}}}{{\varepsilon }_{{\rm{cell}}}{A}_{{\rm{cell}}}}+\frac{1}{{A}_{{\rm{ap}}}{F}_{{\rm{ac}}}}+\frac{1-{\varepsilon }_{{\rm{ap}}}}{{\varepsilon }_{{\rm{ap}}}{A}_{{\rm{ap}}}}}$$

The emissivity of the cell weighted by the spectrum at the aperture temperature is ${\varepsilon }_{{\rm{cell}}}$ (0.15 for the 1.4/1.2 eV tandem and 0.11 for the 1.2/1.0 eV tandem) and the emissivity of the aperture is ${\varepsilon }_{{\rm{a}}{\rm{p}}}\approx 0.1$ .

There is also radiative transfer between the cell and the ambient environment, ${Q}_{{\rm{rad}},{\rm{loss}}}$, but this was found to be negligible at the cell temperature and the calculated view factor between the cell and the environment. Nonetheless, it was included in the calculation of ${Q}_{{\rm{parastic}}}$ for completeness.

Another parasitic heat flow is convective heat loss from the cell to the ambient,

11 $${Q}_{{\rm{c}}{\rm{o}}{\rm{n}}{\rm{v}},{\rm{l}}{\rm{o}}{\rm{s}}{\rm{s}}}=h{A}_{{\rm{c}}{\rm{e}}{\rm{l}}{\rm{l}}}({T}_{{\rm{\infty }}}-{T}_{{\rm{c}}{\rm{e}}{\rm{l}}{\rm{l}}})$$

where $h$ is the convective heat transfer coefficient, and ${T}_{\infty }$ is the ambient temperature. The ambient temperature was measured with a thermocouple, which was blocked from irradiance by the light source using several layers of aluminium foil forming a radiation shield. Ambient temperatures were found to vary between 26 °C at the lowest emitter temperature and 33 °C at the highest emitter temperature. $h$ was calculated using a Nusselt (Nu) correlation for natural convective heat transfer from a horizontal plate at the calculated Rayleigh (Ra) number^63^. Heat transfer coefficients were calculated at each cell/ambient temperature, with the average being $h=5.8\,{\rm{W}}\,{{\rm{m}}}^{-2}\,{{\rm{K}}}^{-1}$.

${Q}_{{\rm{parastic}}}$ is a small and positive quantity at most emitter temperatures. At lower emitter temperatures it is dominated by ${Q}_{{\rm{cond}},{\rm{clips}}}$, whereas at higher emitter temperatures ${Q}_{{\rm{conv}},{\rm{loss}}}$ and ${Q}_{{\rm{rad}},{\rm{gain}}}$ become more important. The potential impact of ${Q}_{{\rm{parastic}}}$ on the efficiency measurement is shown in Extended Data Fig. 6d. Overall, ${Q}_{{\rm{parastic}}}$ has a small impact on the efficiency because ${Q}_{{\rm{parastic}}}$ is two orders of magnitude lower than ${Q}_{{\rm{c}}}$. Because ${Q}_{{\rm{parastic}}}$ is largely derived from modelling and correlation, we do not include it in the efficiency measurement reported. In fact, our calculation of ${Q}_{{\rm{arasitic}}}$ largely predicts a higher efficiency than the measured value, which indicates reported measured efficiency could be conservative.

### Uncertainty propagation

Uncertainty in the efficiency measurement arises from the measurement of ${P}_{{\rm{out}}}$ and the measurement of ${Q}_{{\rm{c}}}$ (equation (1)). From the manufacturer, the calibration accuracy of the HFS is ±3%. We include an additional 10 °C temperature uncertainty in ${T}_{{\rm{s}}}$, the sensor temperature, which comes from the average temperature rise across the sensor as calculated from the thermal resistance of the sensor (4.167 K W^–1^) and the average heat flux passing through the sensor. This leads to an uncertainty of heat absorbed of ${B}_{{Q}_{{\rm{c}}}}=0.03{25Q}_{{\rm{c}}}$. From the source meter, the voltage measurement uncertainty is 0.03% of the voltage (${B}_{v}=(3\times {10}^{-4})V$) and the current measurement uncertainty is 0.06% of the current (${B}_{I}=(6\times {10}^{-4})I$). This leads to an uncertainty in the electric power measurement of ${B}_{P}=\sqrt{{(I\times {B}_{V})}^{2}+{(V\times {B}_{I})}^{2}}$, which is negligible due to the low uncertainty in voltage and current. The absolute uncertainty in measured efficiency, ${B}_{{\eta }_{{\rm{T}}{\rm{P}}{\rm{V}}},{\rm{m}}{\rm{e}}{\rm{a}}{\rm{s}}{\rm{u}}{\rm{r}}{\rm{e}}}$, was calculated as

12 $${B}_{{\eta }_{{\rm{T}}{\rm{P}}{\rm{V}}},{\rm{m}}{\rm{e}}{\rm{a}}{\rm{s}}{\rm{u}}{\rm{r}}{\rm{e}}}=\sqrt{{\left(\frac{{\rm{\partial }}{\eta }_{{\rm{T}}{\rm{P}}{\rm{V}},{\rm{m}}{\rm{e}}{\rm{a}}{\rm{s}}{\rm{u}}{\rm{r}}{\rm{e}}}}{{\rm{\partial }}P}{B}_{P}\right)}^{2}+{\left(\frac{{\rm{\partial }}{\eta }_{{\rm{T}}{\rm{P}}{\rm{V}},{\rm{m}}{\rm{e}}{\rm{a}}{\rm{s}}{\rm{u}}{\rm{r}}{\rm{e}}}}{{\rm{\partial }}{Q}_{{\rm{c}}}}{B}_{{Q}_{{\rm{c}}}}\right)}^{2}}$$

The uncertainty in the model prediction primarily arises from the uncertainty in predicted ${J}_{{\rm{sc}}}$ (${B}_{{J}_{{\rm{sc}}}}\approx 0.03\ast {J}_{{\rm{sc}}}$) from the uncertainty of the EQE measurement of the multi-junction cell, and from the uncertainty of the FTIR reflectance measurement leading to ${B}_{{R}_{{\rm{sub}}}}\approx 0.013$. Propagating these errors through equation (4), the absolute uncertainty in the modelled efficiency, ${B}_{{\eta }_{{\rm{T}}{\rm{P}}{\rm{V}},{\rm{m}}{\rm{o}}{\rm{d}}{\rm{e}}{\rm{l}}}}$, was calculated according to equation (13) and the model uncertainty is shown by the shaded regions in Fig. 3a.

13 $${B}_{{\eta }_{{\rm{T}}{\rm{P}}{\rm{V}},{\rm{m}}{\rm{o}}{\rm{d}}{\rm{e}}{\rm{l}}}}=\sqrt{{\left(\frac{{\rm{\partial }}{\eta }_{{\rm{T}}{\rm{P}}{\rm{V}},{\rm{m}}{\rm{o}}{\rm{d}}{\rm{e}}{\rm{l}}}}{{\rm{\partial }}{J}_{{\rm{s}}{\rm{c}}}}{B}_{{J}_{{\rm{s}}{\rm{c}}}}\right)}^{2}+{\left(\frac{{\rm{\partial }}{\eta }_{{\rm{T}}{\rm{P}}{\rm{V}},{\rm{m}}{\rm{o}}{\rm{d}}{\rm{e}}{\rm{l}}}}{{\rm{\partial }}{R}_{{\rm{s}}{\rm{u}}{\rm{b}}}}{B}_{{R}_{{\rm{s}}{\rm{u}}{\rm{b}}}}\right)}^{2}}$$

The uncertainty in the emitter temperature measurement was calculated from the variation in resistance of the bulb measured at each emitter temperature and the uncertainty in the temperature dependence of the resistance from the literature expression that was used, which is a 0.1% relative error on resistance as a function of temperature^55^. The root mean square of these two yielded temperature measurement uncertainties of less than 4 °C, which had a negligible impact on model uncertainty.

## Online content

Any methods, additional references, Nature Research reporting summaries, source data, extended data, supplementary information, acknowledgements, peer review information; details of author contributions and competing interests; and statements of data and code availability are available at 10.1038/s41586-022-04473-y.

## Figures and Tables

**Extended Data Table 1:**
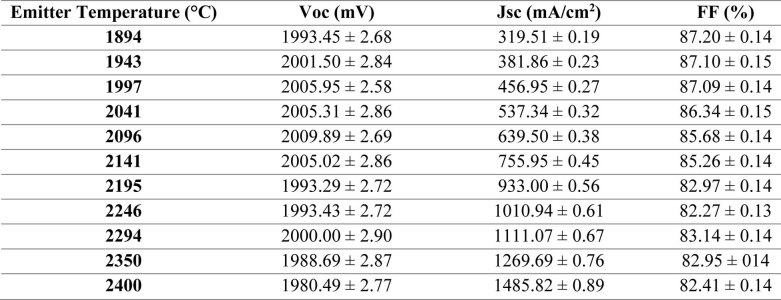
1.4/1.2-eV measurements of ${V}_{{oc}}$, ${J}_{{sc}}$, and ${FF}$

**Extended Data Table 2:**
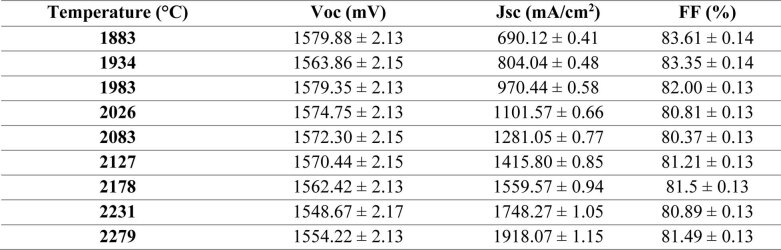
1.2/1.0-eV measurements of ${V}_{{oc}}$, ${J}_{{sc}}$, and ${FF}$

## Data Availability

The data that support the findings of this study are available from the corresponding author upon reasonable request.
